# Genetic characterization of cashmere goat (*Capra hircus*) populations in Mongolia

**DOI:** 10.3389/fgene.2024.1421529

**Published:** 2024-09-17

**Authors:** Sergelen Baldan, Johann Sölkner, Kahsa Tadel Gebre, Gábor Mészáros, Richard Crooijmans, Kathiravan Periasamy, Rudolf Pichler, Bayarjargal Manaljav, Narantuya Baatar, Myagmarsuren Purevdorj

**Affiliations:** ^1^ Department for Animal Science, Mongolian University of Life Sciences, Ulaanbaatar, Mongolia; ^2^ Department of Sustainable Agricultural Systems, University of Natural Resources and Life Sciences, Vienna, Vienna, Austria; ^3^ Department of Animal, Rangeland and Wildlife Sciences (ARWS), Enda-Eyesus Campus, Mekelle University, Mekelle, Ethiopia; ^4^ Wageningen University and Research, Animal Breeding and Genomics, Wageningen, Netherlands; ^5^ Animal Production and Health Laboratory, Joint FAO/IAEA Division of Nuclear Techniques in Food and Agriculture, International Atomic Energy Agency, Vienna, Vienna, Austria

**Keywords:** cashmere goat, phenotypic diversity, genetic diversity, GWAS, Mongolia

## Abstract

**Objective:**

Characterization studies of the phenotypic and genetic diversity of Mongolian goats are limited, despite several goat breeds being registered in the country. This study aimed to evaluate the phenotypic and genetic diversity of 14 cashmere goat populations in Mongolia, consisting largely of identified goat breeds.

**Methods:**

Body weight, cashmere quality, and coat color were the phenotypic traits considered in this study. A linear model was used to fit body weight and cashmere traits, and least squares means (*LSMs*) were estimated for the region and location classes. Genetic diversity and structure were assessed using a goat 50K SNP array.

**Results:**

The studied populations exhibited greater phenotypic diversity at the regional level. A very small overall differentiation index (*Fst*: 0.017) was revealed by Wright’s *Fst* and a very small overall inbreeding index (*F*_*ROH1*_:0.019) was revealed based on runs of homozygosity. Genetic clustering of populations by principal components showed large variances for the two goat populations of the Russian admixture (Gobi Gurvan Saikhan and Uuliin Bor), and smaller but differentiated clusters for the remaining populations. Similar results were observed in the admixture analysis, which identified populations with the highest (Govi Gurvan Saikhan and Uuliin Bor) and lowest (Tsagaan Ovoo Khar) exotic admixtures. A genomewide association study (GWAS) of body weight and cashmere traits identified a few significant variants on chromosomes 2, 4, 5, 9, and 15, with the strongest variant for cashmere yield on chromosome 4. The GWAS on coat color yielded nine significant variants, with the strongest variants located on chromosomes 6, 13, and 18 and potential associations with *KIT*, *ASIP*, and *MC1R* genes. These signals were also found in other studies on coat color and patterns in goats.

**Conclusion:**

Mongolian cashmere goats showed relatively low genetic differentiation and low inbreeding levels, possibly caused by the traditional pastoral livestock management system and the practice of trading breeding bucks across provinces, along with a recent increase in the goat population. Further investigation of cashmere traits using larger samples and alternative methods may help identify the genes or genomic regions underlying cashmere quality in goats.

## 1 Introduction

Cashmere is a fine hair primarily produced by goats from Asian countries, including China, Mongolia, Kazakhstan, Iran, Afghanistan, and India. Depending on its physical properties and origin, goat hair is classified as cashmere, cashgora, mohair, or pashmina hair (Lakshmanan et al., 2016). Goats in Asia, Russia, Australia, and New Zealand produce cashmere hair, with a diameter ranging between 12.5 and 19 µm (Lakshmanan et al., 2016). As a product of high economic importance, cashmere quality directly affects the market value of raw cashmere and has a considerable impact on the income of herders.

Cashmere is one of the top three agricultural export products in Mongolia, which supplies one-third of the world’s raw cashmere (Rysbyek and Lei, 2022). Approximately a quarter of households were considered herders in 2021, and income from cashmere production contributes directly to the livelihoods of herders and those working in the industry (GoM, 2021; Rysbyek and Lei, 2022). In Mongolia, cashmere-related income accounts for up to 70% of the annual income of herders (Meurs et al., 2017). Over the last 3 decades, the total livestock population in the country increased from 25.8 million in 1990 to 67.3 million in 2021, coinciding with the largest growth rate in the goat population, which increased from 5.1 to 26.4 million during the same period (GoM, 2021). This phenomenon puts significant pressure on the country’s semi-dry ecosystem, and overgrazing has been identified as the main contributor to pasture degradation and the decline in vegetation cover (Saizen et al., 2010; Hilker et al., 2014).

Eleven registered Cashmere goat breeds are distributed throughout Mongolia (Samdanjamts and Minjigdorj, 2016). Registered breeds account for approximately 18% of the goat population (Samdanjamts and Minjigdorj, 2016; GoM, 2021). In developing countries, the term ‘breed’ is often used in a broader sense and refers to geographically isolated populations or ethnic groups, rather than phenotypic or production distinction of animals (FAO, 2012).

Population genetics studies have long been used to evaluate the genetic diversity and structures of natural populations (Coates et al., 2009; Helyar et al., 2011; Kumar et al., 2018; Muner et al., 2021). A few descriptors are standardized to define the diversity and admixture levels of populations using different molecular markers. The most common descriptors of population genetic diversity and structure include heterozygosity, differentiation or fixation index (e.g., Wright’s *Fst*), inbreeding level, principal component analysis (PCA) such as multi-dimensional scaling, and admixture analysis (Nicoloso et al., 2015; Colli et al., 2018; Hall, 2022). The information level and comparability of descriptor values vary depending on the molecular markers and origins of the subject population (Coates et al., 2009; Helyar et al., 2011; Putman and Carbone, 2014). Since the 2000s, single nucleotide polymorphism (SNP) has emerged as a cost-effective, reproducible, and reliable tool (Vignal et al., 2002). Currently, many species-specific SNP markers are available at varying densities for numerous models and commercial organisms. The most prominent example of an agricultural species is cattle, with SNP chips ranging from 6 K to 777 K density (Boichard et al., 2012; Illumina Inc.). For goat, the following arrays have been developed: 52K SNP chip (Tosser-Klopp et al., 2014), updated to 65K and GGP 70K arrays by Illumina (GeneSeek^®^ Genomic Profiler™; Illumina Inc.), 10K SNP array IMAGE001v2 (Crooijmans et al., manuscript in preparation) and 50K updated to 60K SNP array (Affymetrix/ThermoFisher Scientific Inc.), and a recently developed 66K array (Qiao et al., 2017) for cashmere goats.

The SNP genotypes offer a wide range of applications, from genetic diversity evaluations to genome-wide association study (GWAS), as well as for the detection of selection signatures across the genome. One limitation of SNP markers is the potential for ascertainment bias, when the SNP discovery was done on a population that is markedly different to the genotyped populations (Geibel et al., 2021). This can affect the results and cause corresponding inferences (Lachance and Tishkoff, 2013).

The phenotypic characterization of Mongolian goats has been limited to the national level. Genetic diversity studies have been conducted using blood protein polymorphisms (Nyamsamba et al., 2003), microsatellites (Nyamsamba et al., 2002; Takahashi et al., 2008; Beketov et al., 2021), mitochondrial DNA (Ganbold et al., 2020; Voronkova et al., 2021), and SNP markers (Mukhina et al., 2022). To our knowledge, limited GWAS of Mongolian cashmere goats, focusing on coat color, have been conducted (Ganbold et al., 2019), and no studies have focused on cashmere production traits, such as body weight or hair quality traits. Herein, we aim to contribute to the characterization of Mongolian Cashmere goats and fill the gap in GWAS for production traits.

This study aimed to describe the phenotypic and genetic diversity of 14 Cashmere goat populations in Mongolia. Additionally, we aimed to identify genomic regions that influence body weight, cashmere quality traits, and coat color.

## 2 Materials and methods

### 2.1 Animals

**FIGURE 1:**
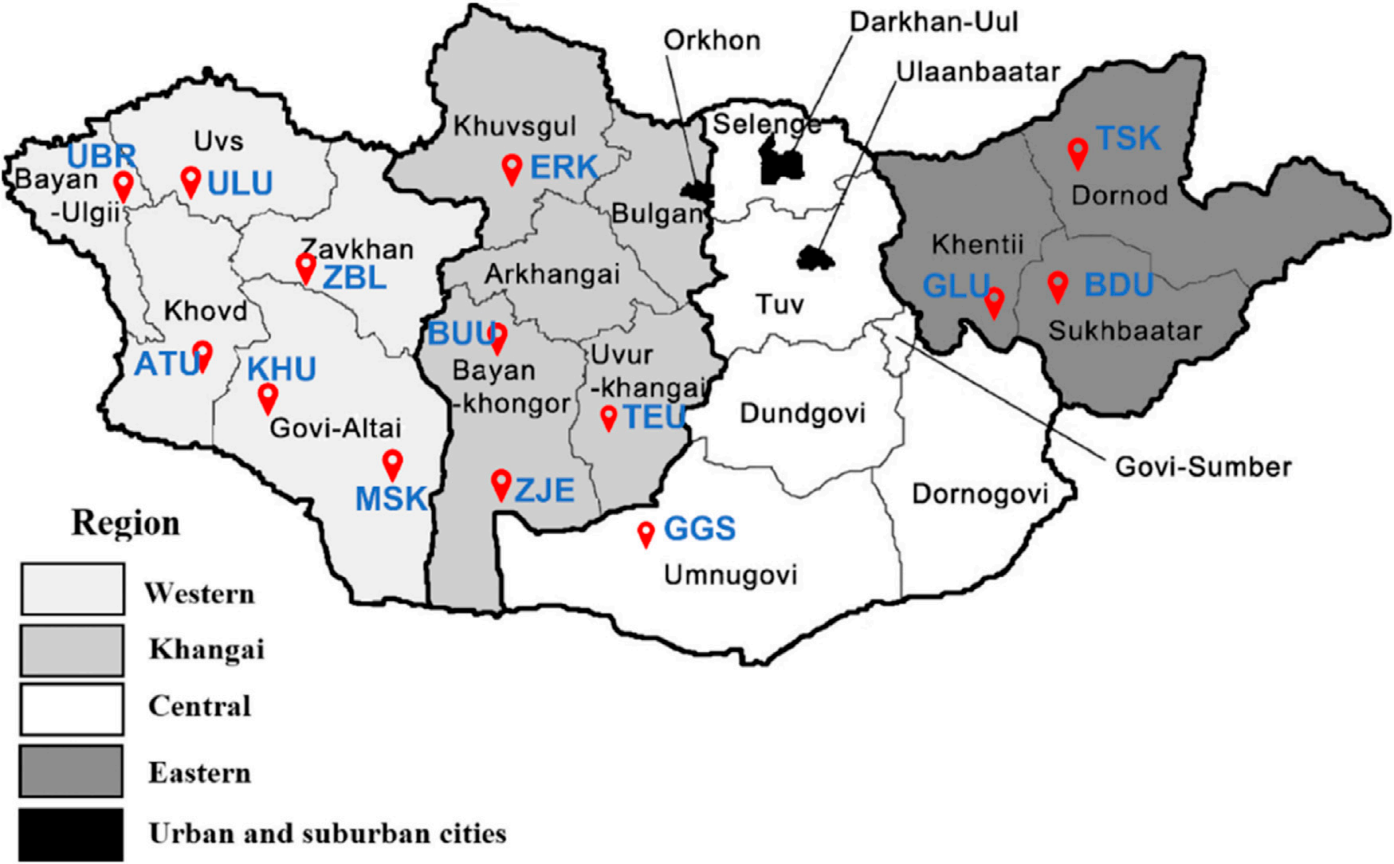
The red markers indicate the distribution of the studied goat populations across Mongolia. Breeds: Alai Ulaan (ATU), Ulgiin Ulaan (ULU), Zavkhan Buural (ZBL), Erchim Khar (ERK), Zalaa Jinst Edren (ZJE), Bayandelger Ulaan (BDU), Govi Gurvan Saikhan (GGS), Uuliin Bor (UBR); Lines (Galshar Ulaan (GLU) and Bumbugur Ulaan (BUU); Local populations: Mungun Sort Khar (MSK), Khuvchiin Ulaan (KHU), Tsagaan Ovoo Khar (TSK) and Teeliin Ulaan (TEU).

Phenotypic measurements were obtained for 2,256 goats from 14 different populations of Cashmere goats in Mongolia. The following traits were measured: body weight, cashmere yield, cashmere diameter, cashmere percentage, cashmere length, and coat color. From the same 14 populations, 1,256 goats were genotyped using an Axiom Goat 60K SNP array. Both phenotypic and genotypic information was available for 537 goats. The distribution of the 14 goat populations studied across the country is shown in Figure 1.

Table 1 shows the origin of each population and the sample sizes for the genotype (N_G_), phenotype (N_P_), and merged (N_M_) datasets. Of the 14 studied populations, 12 were categorized into three socioeconomic regions according to their geographical references. The remaining two populations of Govi Gurvan Saikhan (GGS) and Uuliin Bor (UBR) were categorized into the same ‘region’ called ‘Outliers’ even though they are located in different regions; Govi Gurvan Saikhan in Umnugobi province in the south and Uuliin Bor in Bayan-Ölgii province in the west. Historically, they were developed as the first ‘breeds’ with the introduction of Pri Don and Gorno Altay goat breeds from Russia during the 1960s (Takahashi et al., 2008; Samdanjamts and Minjigdorj, 2016).

**TABLE 1:** The origin and sample sizes of goat populations.

Population name	Population abbreviation	Status	Region^1^	Soum and province of origin	N_P_	N_G_	N_M_
Alai Ulaan	ATU	Breed	West	Altai, Khovd	190	98	25
Khuvchiin Ulaan	KHU	Local	West	Bugat, Govi-Altai	122	99	54
Mungun Sort Khar	MSK	Local	West	Tsogt, Govi-Altai	90	78	-
Ulgiin Ulaan	ULU	Breed	West	Ulgii, Uvs	167	97	39
Zavkhan Buural	ZBL	Breed	West	Dörvöljin, Zavkhan	108	98	60
Bumbugur Ulaan	BUU	Strain	Khangai	Bömbögör, Bayankhongor	172	96	41
Erchim Khar	ERK	Breed	Khangai	Tömörbulag, Khuvsgul	153	95	14
Teeliin Ulaan	TEU	Local	Khangai	Nariinteel, Uvurkhangai	164	70	42
Zalaa Jinst Edren	ZJE	Breed	Khangai	Shinejinst, Bayankhongor	193	83	66
Bayandelger Ulaan	BDU	Breed	East	Bayandelger, Sukhbaatar	176	73	56
Galshar Ulaan	GLU	Strain	East	Galshar, Khentii	289	99	47
Tsagaan Ovoo Khar	TSK	Local	East	Tsagaan-Ovoo, Dornod	157	93	72
Govi Gurvan Saikhan	GGS	Breed	Outliers	Sevrei, Umnugovi	145	98	21
Uuliin Bor	UBR	Breed	Outliers	Bayannuur, Bayan-Ölgii	130	79	-
Overall					2256	1256	537

Socioeconomic regions: West: Bayan-Ölgii, Uvs, Zavkhan, Khovd, and Govi-Altai; Khangai: Khuvsgul, Bulgan, Arkhangai, Bayankhongor, and Uvurkhangai; Central: Selenge, Tuv, Dundgovi, Umnugovi, and Dornogovi; East: Dornod, Khentii and Sukhbaatar.

N_G_, number animals with genotype, N_P_, number animals with phenotype, N_M_, size of the merged set with available genotypes and phenotypes.

Of the studied populations, eight populations were recognized as ‘breed’ (Altai Ulaan (ATU), Ulgiin Ulaan (ULU), Zavkhan Buural (ZBL), Erchim Khar (ERK), Zalaa Jinst Edren (ZJE), Bayandelger Ulaan (BDU), Govi Gurvan Saikhan (GGS), Uuliin Bor (UBR)), two populations had the status of ‘strain/line’ (Galshar Ulaan (GLU) and Bumbugur Ulaan (BUU)), and four populations were local populations without breed or strain status (Mungun Sort Khar (MSK), Khuvchiin Ulaan (KHU), Tsagaan Ovoo Khar (TSK) and Teeliin Ulaan (TEU)).

### 2.2 Phenotype diversity

Phenotypic measurements were collected under the project titled “Improving breed characterization of cashmere goats to facilitate the establishment of the strategic breeding program” No. MON5025 is funded by the International Atomic Energy Agency (IAEA). This project was conducted at the Research Institute of Animal Husbandry (RIAH), Ulaanbaatar, Mongolia, between 2020 and 2022.

Phenotypic traits were measured according to the project protocol. The body weight, coat color, and cashmere yield were recorded in the field during cashmere sampling. Cashmere percentage (i.e., cashmere content), cashmere length, and cashmere diameter were measured according to the “Fiber length and cashmere percentage measuring protocol” at the Fiber Analysis Laboratory in RIAH. Cashmere diameter was measured using a portable OFDA-2000 (Optical-Based Diameter Analyser).

The linear model (Eq. 1) was fitted for quantitative traits considering the animals’ age, sex, environmental factors (both by region or location levels), and each interaction of these variables. Based on the fitted model, least squares means (*LSMs*) were estimated for each trait per region and location within sex and age classes.

$$y_{ijkl}=\mu_{o}+{age}_{i}+{sex}_{j}+{environemnt}_{k}+{age*sex}_{ij}+{age*environment}_{ik}+{sex*environemnt}_{jk}+\varepsilon_{ijkl}$$ (1)

$y_{ijkl}$ - quantitative trait of l individual at age $i\;\left(5\text{ classes}:\text{age classes from }1\text{ to }4,\text{and }5\text{ or over},\text{in years}\right)$ , sex $j\;\left(2\text{ classes}\right)$ , from k region $\left(4\;classes\right)$ ,

$\mu_{o}$ - population mean,

${age*sex}_{ij}$ –the interaction of sex and age

${age*region}_{ik}$ –the interaction of age and environment

${sex*region}_{jk}$ –the interaction of sex and environment

$\varepsilon_{ijkl}$ –an error for l individual, at the age i , sex j , in k environment.

### 2.3 Quality control of genotype data

Quality filtering was performed using PLINK 1.9 (Shaun and Chang, 2005; Chang et al., 2015). Thresholds were applied for the missing genotype rate at 0.05*,* missing individual rate at 0.05, Hardy–Weinberg equilibrium (HWE) deviation <1⨯10^−6^, and minor allele frequency <0.005. When populations were treated separately for quality control, a minor allele frequency <0.05 was applied, while the other filters remained the same.

### 2.4 Genetic structure and diversity

Genetic diversity was defined by the measures of observed (H_*O*_) and expected (H_*E*_) heterozygosity and pairwise *Fst* values among populations. H_*O*_, H_*E*_, and pairwise *Fst* values were obtained using PLINK 1.9. Genetic structure was evaluated by PCA (Bradburd et al., 2016) and admixture plots. Admixture analysis was performed using ADMIXTURE v1.3.0 software (Alexander et al., 2009). The SNPs were pruned at *r* ^2^ > 0.1 using PLINK 1.9 before admixture analysis. The CV error estimates were reported using the Admixture software with*--cv* command. Admixture and PCA results were visualized using *R*. The package *detectRUNS* (Biscarini et al., 2019) in *R* was used to quantify the runs of homozygosity (ROH) segments. *ConsecutiveRuns* method was applied with the following parameters: minimum number of 20 homozygous SNP, minimum lengths at 1 Mb (or 2, 4, 8, and 16 Mb), number of heterozygous allowed 0, maximum number of missing SNP 2, and maximum gap between two SNPs 1 Mb. The inbreeding coefficient (*F*_*ROHk*_) based on ROH was estimated using Eq. 2, where $\sum_{ROHk}length$ is the sum of ROH lengths at $\dot{k}$ minimum length and $L_{autosome}$ is the total length of the autosomal genome. The autosomal genome was approximately 2.46 Gb.

$$F_{ROHk}=\frac{\sum_{k}length\left(ROH\right)}{L_{autosome}}$$ (2)

### 2.5 Genome-wide association study (GWAS)

For the GWAS, SNPs were filtered with thresholds of MAF<0.05, HWE *p*-value < 1⨯10^−6^, missingness rate 0.1, and genotyping rate 0.1. Autosomal variants (35,284 SNPs, Supplementary Table S1) that passed quality filtering were used for analysis. Five quantitative traits (body weight, cashmere percentage, cashmere length, cashmere diameter, and cashmere yield), and one qualitative trait (coat color) were tested for genome association using GCTA v1.94.1 software (Yang et al., 2011). For quantitative traits, age was considered a quantitative covariate (*--qcovar*), sex and region were considered a categorical covariates (*--covar*) (Eq. 3). The model is defined as follows:

$$y=\mu +\left(x_{c\left(t\right)}*b_{c\left(t\right)}\right)+\left(x_{q\left(1\right)}*b_{q\left(1\right)}\right)+\left(x_{p\left(1\right)}*b_{p\left(1\right)}\right)+g+e$$ (3)

y : quantitative trait

μ : mean term

x in $x_{c\left(t\right)}$ : presence (1) or absence (0) of the categorical variable

$c\left(t\right)$ : categorical variable level (2 for sex)

$b_{c\left(t\right)}$ : difference in mean for the categorical level

x in $x_{q\left(1\right)}\;and\;x_{p\left(1\right)}$ : presence (1) or absence (0) of a quantitative variable

$q\left(1\right)$ : value of the quantitative variable (1 – 7 for age)

$b_{q\left(1\right)}$ : difference in mean for quantitative variables at 1

$p\left(1\right)$ : value of the quantitative variable (1–12 for region)

$b_{p\left(1\right)}$ : difference in mean for quantitative variables at 1 g : genetic value

e : residual

For qualitative traits, the linear mixed model was as follows (Eq. 4):

y=μ+g+e (4)

y : qualitative trait

μ : mean term

g : genetic value

e : residual

The proportion of variance explained by SNPs (SNP-based heritability or $\hat{h}$ ^2^_SNP_) for each trait was estimated using the*--reml* command in GCTA. The population structure correction based on the genomic relationship matrix was included by GCTA. To avoid Type I errors, false discovery rate (FDR) and Bonferroni correction methods were used (Benjamini and Hochberg, 1995; Weisstein, 2004). The common significance thresholds for all breeds were set according to Bonferroni correction at 0.05 (0.05/35,284), and a ‘suggestive *p*-value’ (1/35,284) as proposed by Lander and Kruglyak (1995). Manhattan and QQ plots were obtained using the *qqman* package in *R* (Turner, 2014).

Locations of significant variants were annotated based on the ARS1.2 (GCF_001704415.2) goat assembly, as available on the NCBI website.

## 3 Results

### 3.1 Phenotype diversity

Linear models for quantitative traits were fitted considering both location and regional levels as environmental classes. The model’s adjusted *R-squared* values ranged between 0.154 and 0.666, with the highest explained variance for body weight (0.666) and the lowest explained variance for cashmere length (0.154). The *p-*values (<2.2e-16) of the model for the five traits were all significant, suggesting high or large dependence on the considered factors. The model fit is also indicated by the *F* value and its significance level for different factors and their interactions. For example, an animal’s body weight is largely age-dependent, with an *F* value of 2,384.95 while cashmere length varies the least by sex, with an *F* value of 1.43.

The *LSMs* and their significance levels were more distinct at the regional level and were split according to sex (Table 2). Regarding body weight, all females and males within the regions were significantly different from each other, except in two cases. The two cases that did not show significant differences in body weight were males in the West (ATU, KHU, MSK, ULU and ZBL) and Outlier (GGS, UBR) regions. Also, males in Khangai (BUU, ERK, TEU and ZJE) and females in the West region (ATU, KHU, MSK, ULU and ZBL) did not show significant difference in body weight (Table 2). Female goats in the Khangai region (BUU, ERK, TEU and ZJE) were the smallest in size, followed by female goats in the West region (ATU, KHU, MSK, ULU and ZBL). Females in Outlier regions (GGS, UBR) showed the highest body weight, followed by those in the East region (BDU, GLU, TSK). Male goats in the West (ATU, KHU, MSK, ULU and ZBL) and Outlier (GGS, UBR) regions showed the highest body weight, followed by males in the East (BDU, GLU, TSK) and then in the Khangai region (BUU, ERK, TEU and ZJE). Overall, goats in the Outlier regions (GGS, UBR) had the highest, whereas goats in the Khangai region (BUU, ERK, TEU and ZJE) had the lowest body weight.

**TABLE 2:** The least square means (*LSM ± SE*) for traits over regions.

Region	Sex	N	BW (kg)	CaP (%)	CaL (cm)	CaD (µm)	CaY (g)
East	Female	280	29.2 ± 0.41^c^	88.5 ± 0.59^f^	4.67 ± 0.056^a^	15.9 ± 0.056^bcd^	385 ± 6.45^a^
	Male	342	33.1 ± 0.39^d^	84.6 ± 0.54^e^	4.85 ± 0.051^ab^	16.0 ± 0.051^cd^	391 ± 5.86^a^
Khangai	Female	367	22.2 ± 0.35^a^	79.5 ± 0.51^d^	5.35 ± 0.048^d^	15.8 ± 0.049^abc^	377 ± 5.46^a^
	Male	315	26.9 ± 0.43^b^	75.6 ± 0.57^bc^	5.13 ± 0.054^cd^	16.1 ± 0.055^d^	454 ± 6.11^b^
West	Female	391	27.2 ± 0.34^b^	77.7 ± 0.50^cd^	4.98 ± 0.047^bc^	15.6 ± 0.048^a^	460 ± 5.33^b^
	Male	286	37.6 ± 0.44^e^	75.2 ± 0.60^ab^	5.11 ± 0.056^c^	15.7 ± 0.057^ab^	532 ± 6.37^c^
Outliers	Female	109	33.7 ± 0.69^d^	73.7 ± 0.94^ab^	5.75 ± 0.088^e^	17.7 ± 0.075^e^	534 ± 13.68^cd^
	Male	166	36.7 ± 0.56^e^	72.2 ± 0.78^a^	5.93 ± 0.074^e^	17.9 ± 0.089^e^	574 ± 11.40^d^

BW, body weight; CaP, cashmere percentage; CaL, cashmere length; CaD, cashmere diameter; CaY, cashmere yield.

Letters (a-e) indicate significant differences in a trait within regions and sex classes. Same letters imply no significant difference while different letters indicate observed significant differences within regions and sex classes.

For the cashmere percentage, the overall estimates ranged from 72.2% to 88.5% across regions. Goats in the Outlier regions had the lowest cashmere percentage while the goats in the East region had the highest cashmere content. The mean cashmere length ranged from 4.67 to 5.93 cm, with the length being the smallest in the East and largest in the Outlier populations. A similar trend was observed for cashmere yield, with the least yields for goats (males and females) in the East and females in the Khangai region and the greatest yields for goats in the Outlier populations (GGS, UBR). The cashmere (fibers) diameter ranged from 15.6 to 17.9 µm. Goats in the West region had the finest cashmere, whereas those in the Outlier regions had the thickest hair.

*LSMs* estimated for the age and sex classes are listed in Table 3. The strongest sexual dimorphism was observed for body weight, with differences increasing with age. Among cashmere traits, the most significant difference was observed in cashmere yield, whereas cashmere length and diameter showed the least differences between sex classes within the same age. The cashmere percentages were higher in females aged over 2 years, but the difference was weak.

**TABLE 3:** The *LSM* estimates of quantitative traits over age and sex classes of goat populations.

Traits	Sex	N	Age 1	N	Age 2	N	Age 3	N	Age 4	N	Age ≥5
BW (kg)	F	225	18.0 ± 0.54^a^	224	22.8 ± 0.52^b^	159	28.5 ± 0.61^d^	235	29.4 ± 0.50^d^	304	32.9 ± 0.44^e^
	M	252	18.8 ± 0.56^a^	250	25.4 ± 0.52^c^	160	33.4 ± 0.63^e^	183	38.1 ± 0.59^f^	264	46.3 ± 0.48^g^
CaP (%)	F	225	73.5 ± 0.67^a^	224	84.8 ± 0.70^d^	159	81.7 ± 0.83^cd^	235	81.7 ± 0.68^cd^	304	81.3 ± 0.60^c^
	M	252	73.6 ± 0.70^a^	250	80.5 ± 0.66^c^	160	78.6 ± 0.83^bc^	183	77.1 ± 0.78^b^	264	79.6 ± 0.65^bc^
CaL (cm)	F	225	4.95 ± 0.07^abc^	224	4.84 ± 0.06^a^	159	5.17 ± 0.08^bcd^	235	5.27 ± 0.06^d^	304	5.32 ± 0.06^d^
	M	252	4.82 ± 0.06^a^	250	4.94 ± 0.06^ab^	160	5.27 ± 0.08^cd^	183	5.36 ± 0.07^d^	264	5.43 ± 0.06^d^
CaD (µm)	F	225	15.4 ± 0.07^ab^	224	15.8 ± 0.08^bc^	159	16.1 ± 0.09^cd^	235	16.2 ± 0.07^d^	304	16.4 ± 0.06d^e^
	M	252	15.2 ± 0.07^a^	250	15.8 ± 0.07^c^	160	16.3 ± 0.09^de^	183	16.6 ± 0.08^e^	264	17.2 ± 0.07^f^
CaY (gr)	F	225	380 ± 8.02^a^	224	363 ± 8.37^a^	159	434 ± 9.41^b^	235	431 ± 7.76^b^	304	485 ± 7.17^c^
	M	252	421 ± 7.66^b^	250	396 ± 7.91^ab^	160	491 ± 9.41^c^	183	507 ± 8.80^cd^	264	534 ± 8.23^d^

BW, body weight; CaP, cashmere percentage; CaL, cashmere length; CaD, cashmere diameter; CaY, cashmere yield.

Letters (a-f) indicate significant differences (<0.05) of a trait within sex and age classes.

*Sex: F, female and M, male.*

The cashmere percentage was the highest for both sex classes at 2 years and remained steady without a distinct difference thereafter.

### 3.2 Genetic structure and diversity

#### 3.2.1 Quality control of genotype data

The Axiom Goat 50K SNP array contains 58,655 markers, of which 41,007 were successfully genotyped in the samples. A total of 1,106 animals and 38,343 variants passed quality control, and 36,887 of them were autosomal (Supplementary Table S1). In total, 62.8% of the Goat 50K SNP array markers were informative for the current dataset.

#### 3.2.2 Principal component analysis

**FIGURE 2:**
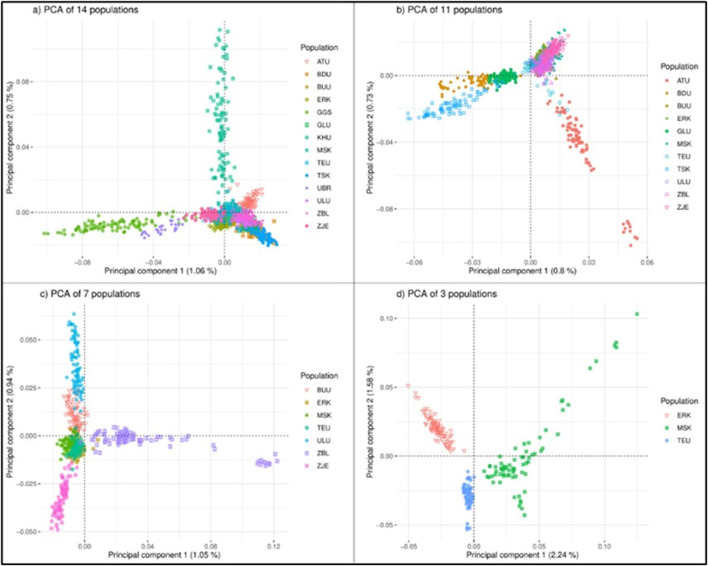
PCA analysis of the goat populations considering different sets of populations: **(A)** 14 populations, **(B)** 11 populations, **(C)** 7 populations **(D)** 3 populations. Breeds: Alai Ulaan (ATU), Ulgiin Ulaan (ULU), Zavkhan Buural (ZBL), Erchim Khar (ERK), Zalaa Jinst Edren (ZJE), Bayandelger Ulaan (BDU), Govi Gurvan Saikhan (GGS), Uuliin Bor (UBR); Lines (Galshar Ulaan (GLU) and Bumbugur Ulaan (BUU); Local populations: Mungun Sort Khar (MSK), Khuvchiin Ulaan (KHU), Tsagaan Ovoo Khar (TSK), and Teeliin Ulaan (TEU).

Clustering based on the principal components for all 14 populations showed that all populations were dispersed in approximately three rays (Figure 2A). The first noticeable direction was the group of Outlier populations (GGS, UBR), the second ray was the population of KHU (local population), and the remaining populations were clustered together as one cluster with few sparks. In each PCA plot from A) to D) in Figure 2, dispersed populations were excluded to zoom in and observe the appearance of remaining populations in more detail. This approach created four PCA plots: A) with 14 populations, B) with 11 populations (GGS, UBR, and KHU were excluded), C) with seven populations after excluding TSK, GLU, and BDU in the East region in addition to those discarded in Figures 2B–D) three populations excluding ULU, ZJE, ZBL, and BUU in addition to those excluded in Figures 2B, C. The outward behavior of KHU was not expected, as this population is neither a breed nor a strain. After removing three populations from the first plot, the remaining 11 populations clustered as the ‘East’ populations, the cluster of Altain Ulaan, and the rest (Figure 2B). Outward populations (ATU and three populations in the East region) were removed to zoom in, leaving seven populations in the plot. The seven populations were separately dispersed as ZBL, ZJE, and ULU, leaving overlapping clusters of three populations in the middle (Figure 2C). The outgoing populations were removed again, and the final PCA was obtained for three populations: ERK, MSK, and TEU (Figure 2D). The three populations remaining on the last multi-dimensional scale were not geographically close to each other, originating from three different provinces in north Khuvsgul (ERK), southwest Gobi-Altai (MSK), and the central region of Khangai, Uvurkhangai (TEU).

When all populations were considered, only 1.8% (PC1: 1.06%, PC2: 0.75%) of the total variance explained the clustering based on the first two principal components. When populations were reduced to 11 and 7, the percentages of total variance remained very small, giving PC1 values of 0.8% and 1.05%, for 11 and 7 populations, respectively. For three-population clustering (Figure 2D), the cumulative percentage of total variation was 3.82 (PC1: 2.24%, PC2: 1.58%).

Populations are displayed within regional classes on the PCA plot in Figure 2 (Supplementary Material). The cumulative percentages of the total variances in the regional PCA were 2.8%, 3.34%, 4.3%, and 7.76% for the Khangai, West, East, and Outlier regions, respectively. In the PCA plot for the West region, KHU and ATU clustered outward from the other three populations. Population ATU is a breed registered in 2016, whereas KHU is a local population. Among the remaining three populations, ZBL and ULU are registered as goat breeds, whereas MSK is a local population. In the Khangai region, the BUU, ZJE, and ERK populations were dispersed outward, leaving TEU in the middle. Interestingly, BUU, ZJE, and ERK are ‘breeds’ (ERK, ZBL) and ‘strains’ (BUU) while TEU is an indigenous/local population. The East region showed three distinct clusters, but GLU and BDU were connected at one end, which is in line with their closer geographical proximity to the TSK population. Of these three, TSK is local, whereas GLU and BDU are a strain and breed, respectively. Outlier populations showed the largest percentage of variation (PC1: 4.67%; PC2: 3.09%) and were clustered separately; however, GGS showed two separate directions on PC2.

#### 3.2.3 Pairwise *Fst*

The overall mean pairwise *Fst* value was 0.017 across 14 populations, ranging from 0.007 (TEU-BUU) to 0.044 (TSK-UBR) (Table 4). The population that showed the largest mean pairwise *Fst* was UBR, with a mean pairwise *Fst* of 0.039, the most pronounced differentiation with TSK (0.044), and the least with ERK (0.034). The UBR and TSK populations are located on the western and eastern ends of the country, respectively, and the largest differentiation index between them was in line with their geographical distances. The second largest mean differentiation index was observed for GGS, with an average pairwise *Fst* of 0.023.

**TABLE 4:** Pairwise *Fst* values between pairs of goat populations.

	ATU	BDU	BUU	ERK	GGS	GLU	KHU	MSK	TEU	TSK	UBR	ULU	ZBL	ZJE
ATU	-													
BDU	0.018	-												
BUU	0.013	0.014	-											
ERK	0.015	0.016	0.011	-										
GGS	0.025	0.026	0.018	0.020	-									
GLU	0.013	0.009	0.009	0.011	0.022	-								
KHU	0.019	0.022	0.016	0.018	0.025	0.017	-							
MSK	0.017	0.018	0.012	0.014	0.023	0.013	0.019	-						
TEU	0.012	0.013	0.007	0.010	0.015	0.01	0.015	0.011	-					
TSK	0.018	0.017	0.015	0.016	0.027	0.013	0.022	0.019	0.014	-				
UBR	0.042	0.043	0.036	0.034	0.037	0.039	0.043	0.040	0.034	0.044	-			
ULU	0.015	0.017	0.009	0.012	0.024	0.011	0.019	0.015	0.012	0.017	0.039	-		
ZBL	0.015	0.016	0.011	0.013	0.024	0.012	0.019	0.015	0.011	0.017	0.040	0.013	-	
ZJE	0.017	0.018	0.012	0.014	0.019	0.013	0.019	0.015	0.011	0.019	0.038	0.016	0.015	-

Breeds: Alai Ulaan (ATU), Ulgiin Ulaan (ULU), Zavkhan Buural (ZBL), Erchim Khar (ERK), Zalaa Jinst Edren (ZJE), Bayandelger Ulaan (BDU), Govi Gurvan Saikhan (GGS), Uuliin Bor (UBR); Lines: (Galshar Ulaan (GLU) and Bumbugur Ulaan (BUU); Local populations: Mungun Sort Khar (MSK), Khuvchiin Ulaan (KHU), Tsagaan Ovoo Khar (TSK) and Teeliin Ulaan (TEU).

#### 3.2.4 Admixture

**FIGURE 3:**
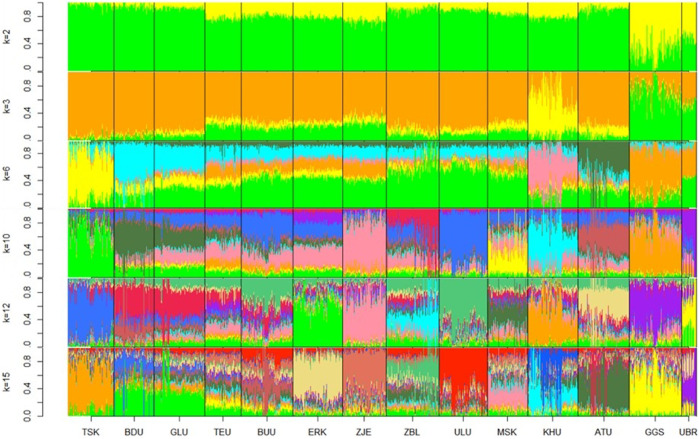
Admixture plots at ancestry numbers k = 2, 3, 6, 10, 12, and 15 for goat populations. The optimal k value was 15. Breeds: Alai Ulaan (ATU), Ulgiin Ulaan (ULU), Zavkhan Buural (ZBL), Erchim Khar (ERK), Zalaa Jinst Edren (ZJE), Bayandelger Ulaan (BDU), Govi Gurvan Saikhan (GGS), Uuliin Bor (UBR); Lines (Galshar Ulaan (GLU) and Bumbugur Ulaan (BUU); Local populations: Mungun Sort Khar (MSK), Khuvchiin Ulaan (KHU), Tsagaan Ovoo Khar (TSK) and Teeliin Ulaan (TEU).

Admixture plots were obtained for k values ranging from 2 to 20; however, only selected plots (k = 2, 3, 6, 10, 12, and 15) are shown in Figure 3. Starting from ancestry number 2, populations Govi Gurvan Saikhan and Uuliin Bor showed the largest percentage of the second ancestry, in line with the PCA clustering results that are assumed to have an admixture from outside the country. At k = 2, the populations also showed ‘regional’ differences, where the East populations contained the largest proportion of first ancestry, and populations in the Outlier group showed the lowest proportions of first ancestry. The Khuwchiin Ulaan population showed the largest proportion of second ancestry at k = 3, which is consistent with the results of the PCA (Figure 3). At k = 6, different ancestries were observed for TSK, KHU, and ATU (apart from GGS and UBR), whereas the remaining nine populations showed mixed ancestries with less differentiation. With k = 10, ZJE and ULU showed relatively differentiated admixtures. While most populations showed unique ancestry with ancestry k = 12, populations BDU and GLU (East region) and populations TEU and BUU (Khangai region) showed the least distinction within each pair. The proportions of various ancestries did not show noticeable differences between breeds and indigenous goats, suggesting that genomic differentiation among populations may primarily be due to geographical isolation rather than selection pressure.

Based on the lowest cross-validation error (Supplementary Information Table 5) The optimal number of ancestries was k = 15, suggesting a potential subpopulation within one population.

**TABLE 5:** The linear model parameters for quantitative traits of goat populations.

Traits	Adj-R^2^	*F* Statistic	*p*-value	*F* Value					
				Age	Sex	Region	Age and sex	Age and Region	Region and sex
Body weight	0.666	334.7	<2.2e-16	2384.95***	537.19***	212.37***	246.19**	39.74***	30.53***
Cashmere percentage	0.215	52.3	<2.2e-16	49.84***	37.79***	172.05***	0.57	6.22***	1.47
Cashmere length	0.154	35.0	<2.2e-16	103.28***	1.43	228.3***	2.27	8.43***	6.11***
Cashmere diameter	0.451	155.1	<2.2e-16	689.82***	42.25***	347.21***	44.55***	8.5***	5.63***
Cashmere yield	0.356	97.97	<2.2e-16	397.20***	123.81***	163.36***	8.64**	38.04***	13.94***

The “and” implies the interaction between two factors. For example, “Age and Sex” indicates the interaction between age and sex factors.

Asterisks on F values, “**” and “***” indicate the significance level at 0.01 and 0.001 *p*-value, respectively.

#### 3.2.5 Inbreeding levels based on runs of homozygosity

The ROH segments were detected at minimum lengths of 1, 2, 4, 8, and 16 Mb. Among all 14 populations, Uuliin Bor showed consistently high inbreeding levels, with *F*_*ROH1*_ = 0.053 ± 0.068 and *F*_*ROH16*_ = 0.018 ± 0.044. Details of the inbreeding coefficients at different *ROH* segments of the studied goat populations are presented in Table 6. The relatively high inbreeding coefficient for the UBR could be attributed to the small sample size (N = 26). The populations with the second and third largest genomic inbreeding coefficient *F*_*ROH1*_ were GGS (0.029 ± 0.030) and TSK (0.028 ± 0.045).

**TABLE 6:** Inbreeding coefficients on different minimum ROH segments for goat populations.

Population	*F*_*ROH1*_	*F*_*ROH2*_	*F*_*ROH4*_	*F*_*ROH8*_	*F*_*ROH16*_
ATU	0.017 ± 0.016	0.013 ± 0.016	0.008 ± 0.014	0.004 ± 0.012	0.001 ± 0.007
BDU	0.023 ± 0.031	0.018 ± 0.029	0.012 ± 0.025	0.006 ± 0.019	0.003 ± 0.013
BUU	0.014 ± 0.027	0.012 ± 0.027	0.010 ± 0.026	0.007 ± 0.021	0.004 ± 0.012
ERK	0.013 ± 0.008	0.010 ± 0.008	0.007 ± 0.007	0.003 ± 0.005	0.001 ± 0.003
GGS	0.029 ± 0.030	0.025 ± 0.027	0.019 ± 0.027	0.011 ± 0.021	0.004 ± 0.010
GLU	0.009 ± 0.006	0.007 ± 0.006	0.004 ± 0.005	0.002 ± 0.004	0.001 ± 0.003
KHU	0.022 ± 0.015	0.018 ± 0.014	0.013 ± 0.012	0.007 ± 0.009	0.002 ± 0.005
MSK	0.017 ± 0.031	0.014 ± 0.030	0.011 ± 0.026	0.007 ± 0.019	0.001 ± 0.005
TEU	0.010 ± 0.012	0.008 ± 0.012	0.006 ± 0.011	0.003 ± 0.009	0.001 ± 0.005
TSK	0.028 ± 0.045	0.024 ± 0.045	0.020 ± 0.043	0.014 ± 0.038	0.007 ± 0.024
UBR	0.053 ± 0.068	0.048 ± 0.068	0.040 ± 0.068	0.030 ± 0.064	0.018 ± 0.044
ULU	0.018 ± 0.020	0.015 ± 0.019	0.010 ± 0.017	0.005 ± 0.012	0.002 ± 0.005
ZBL	0.015 ± 0.025	0.012 ± 0.025	0.008 ± 0.022	0.005 ± 0.016	0.001 ± 0.006
ZJE	0.018 ± 0.019	0.015 ± 0.019	0.011 ± 0.017	0.006 ± 0.015	0.002 ± 0.008
mean	0.019 ± 0.027	0.016 ± 0.026	0.011 ± 0.024	0.007 ± 0.020	0.003 ± 0.012

Breeds: Alai Ulaan (ATU), Ulgiin Ulaan (ULU), Zavkhan Buural (ZBL), Erchim Khar (ERK), Zalaa Jinst Edren (ZJE), Bayandelger Ulaan (BDU), Govi Gurvan Saikhan (GGS), Uuliin Bor (UBR); Lines: (Galshar Ulaan (GLU) and Bumbugur Ulaan (BUU); Local populations: Mungun Sort Khar (MSK), Khuvchiin Ulaan (KHU), Tsagaan Ovoo Khar (TSK) and Teeliin Ulaan (TEU).

Of the 14 populations, nine had relatively low *F*_*ROH1*_ (<0.02), whereas the remaining five populations (BDU, GGS, TSK, and UBR) had *F*_*ROH1*_ > 0.02. There was no marked difference between the inbreeding coefficients of the ‘breed’ and ‘local’ populations.

#### 3.2.6 Heterozygosity and *F*_*SNP*_

The observed and expected heterozygosity (H_*E*_ and H_*O*_) and *F*_*SNP*_ were estimated for two different cases. First, all 14 populations were considered as one large population, ignoring their substructures; second, the 14 populations were treated separately, applying population-specific quality control. For the first case, the overall H_*E*_ *,* H_*O,*_ and F_*SNP*_ values were 0.386 ± 0.0003, 0.380 ± 0.014, and 0.014 ± 0.037, respectively (Supplementary Material; Table 5).

When populations were considered independently, *F*_*SNP*_ ranged between −0.002 (GGS) and −0.020 (UBR). For H_*E*_ and H_*O*_, GGS showed the highest heterozygosity (H_*E*_ = 0.4035, H_*O*_ = 0.4027), TSK showed the smallest expected heterozygosity, and ATU and UBR showed the smallest observed heterozygosity (both H_*O*_ = 0.3933). Although the separate inbreeding coefficients (*F*_*SNP*_) appeared negative, relatively large standard deviations suggest the presence of inbred individuals within populations.

#### 3.2.7 Genome-wide association study

##### 3.2.7.1 Quantitative traits

GWAS was performed for five quantitative traits: body weight, cashmere percentage, cashmere length, cashmere diameter, and cashmere yield. The estimated $\text{SNP}‐\text{based heritabilities }\left({\hat{h}}_{SNP}^{2}\right)$ were as follows: body weight: 0.782 ± 0.072, cashmere percentage: 0.773 ± 0.078, cashmere length: 0.712 ± 0.098, cashmere diameter: 0.682 ± 0.109 and cashmere yield: 0.734 ± 0.076.

No significant variants were detected in the five quantitative traits, although a few variants passed the suggested threshold. The suggested variants are listed in Table 7. The suggestive SNP associated with body weight in our study was located on chromosome 5, approximately 3 Mb upstream of the *CRADD* (chr5:23.3–23.4Mb) gene. The suggestive SNP for cashmere diameter was located on chromosome 9 at position 28,641,101, which is closely located to the protein coding gene *ARMC2* (Armadillo Repeat Containing 2) at position 28,486,971–28,647,051. The *ARMC2* gene is involved in the sperm flagellar axoneme and its function. There were no genes in the region for cashmere percentage, cashmere length, and cashmere yield SNP.

**TABLE 7:** SNP variants observed on GWAS for quantitative traits of goat populations.

Trait	SNP name	Location	A1/A2	*p*-value	FDR	MAF
Body weight	AX-123311737	5: 20,015,454	T/C	2.6731e-05	0.943	0.129
Cashmere percentage	AX-123321177	5: 108,420,020	A/C	2.3849e-05	0.842	0.413
Cashmere length	AX-413837400	2: 12,548,235	G/C	1.5836e-05	0.559	0.068
	AX-123297114	15: 10,429,208	A/G	1.3154e-05	0.464	0.158
Cashmere diameter	AX-385269016	9: 28,641,101	A/G	1.9704e-05	0.695	0.322
Cashmere yield	AX-123322462	4: 6,418,954	T/C	4.9915e-06	0.176	0.367

##### 3.2.7.2 Coat color

GWAS focusing on coat color, considering each pair of colors (white vs. red and white vs. black), are presented in Table 8.

**TABLE 8:** Significant SNPs associated with coat color of goat populations.

Case vs. control	SNP name	Location	A1/A2	*p*-value	FDR	MAF
White vs. red						
	AX-123294167	18: 16,155,381	C/T	1.15073e-18	0.000	0.267
	AX-123312274	18: 16,198,866	G/A	6.09811e-15	0.000	0.376
	AX-123262623	24: 48,117,628	G/A	1.14205e-09	0.000	0.057
	AX-123307776	13: 60,571,963	T/C	1.80278e-08	0.001	0.203
	AX-519549237	6: 66,864,627	A/G	4.96117e-08	0.002	0.126
	AX-123254476	13: 61,538,529	G/A	1.61715e-07	0.006	0.271
	AX-123266901	2: 76,537,098	G/A	2.06105e-07	0.007	0.148
	AX-123303762	1: 114,546,836	G/A	2.13429e-07	0.008	0.277
White vs. black						
	AX-123294167	18: 16,155,381	C/T	1.24571e-07	0.004	0.386

**FIGURE 4:**
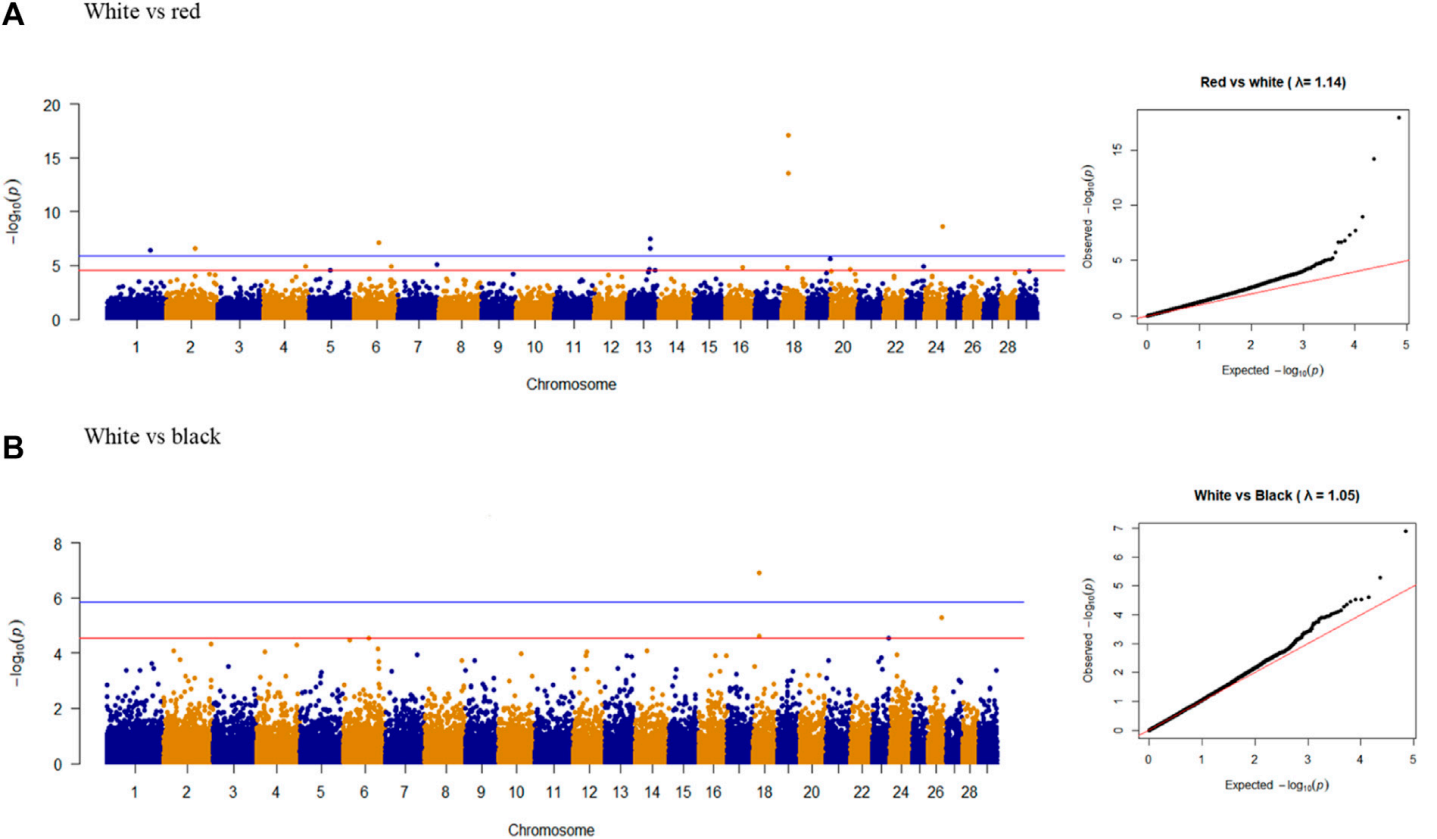
Manhattan plots and Q-Q plots of genome-wide association study for **(A)** white vs. red and **(B)** white vs. black colors in goat populations.

Nine overlapping significant variants were identified for coat color (Figure 4), of which eight variants were unique and distributed on chromosomes 1, 2, 6, 13, 18, and 24. The significant locus identified at 48.11 Mb on chromosome 24 was located upstream of *MC5R* (24:43.86–43.87 Mb) and *MC2R* (18:43.89–43.91 Mb) genes and downstream of *MC4R* gene (24:59.345–59.347 Mb) in the goat genome. There were no significant variants associated with red vs. black coat color.

The closest genes with a possible influence on ‘pigmentation’ were identified for each significant variant (Table 9). For example, the variant on chromosome 1 was located between *GMPS* (111.2–111.3 Mb) and *HPS3* (118.5–118.6 Mb) genes, while the variant on chromosome 2, was flanked by *UBXN4* (74.61–74.64 Mb) and *ZEB2* (84.0–84.1 Mb) pigmentation genes. The variant on chromosome 6 was located within the *CORIN* gene (66.8–67.1 Mb), which was listed on the pigmentation gene catalog, and described to influence coat color dilution. The *KIT* gene, a strong candidate related to pigmentation was located on chromosome 6, at 70.71–70.79 Mb, downstream of an identified variant on chromosome 6.

**TABLE 9:** Pigmentation genes around the observed variants.

SNP name	Chromo-some	Locus, Mb	Genes harboring the locus	Coat color candidate genes in the proximity
AX-123303762	1	114.54	Intergenic region	*GMPS, HPS3*
AX-123266901	2	76.53	*THSD7B*	*UBXN4, ZEB2*
AX-519549237	6	66.86	*CORIN*	*CORIN, KIT*
AX-123307776	13	60.57	*BCL2L1*	*TFAP2C, POFUT1*
AX-123254476	13	61.53	*MAPRE1*	*TFAP2C, POFUT1*
AX-123294167	18	16.15	*LOC102181419*	*FANCA, SPIRE2*
AX-123312274	18	16.19	*GAS8*	*FANCA, SPIRE2*
AX-123262623	24	48.11	*ZBTB7C*	*MC2R, MC4R, IER3IP1*

The two variants on chromosome 13 were located between the *BCL2L1* (60.52–60.58 Mb) and *MAPRE1* (60.52–60.58 Mb) genes. *BCL2L1* could be an ortholog of the *BCL2* ‘pigmentation’ gene. In the goat genome, *BCL2L1* has been described to play a role in apoptosis regulation, according to the Gene Ontology database by Ensembl. The nearest ‘pigmentation’ genes on chromosome 13 were *TFAP2C* (58.65–58.66 Mb) and *POFUT1* (61.0–61.02 Mb). The two of the most frequently mentioned genes in GWAS on goat coat color, *ASIP* (63.22–63.24 Mb), and *AHCY* (63.26–63.27 Mb) were located on chromosome 13, which were downstream of two observed variants in our study. Two other significant variants on chromosome 18 were in *LOC102181419* and *GAS8*, with the nearest ‘pigmentation’ genes being *FANCA* (16.0–16.04 Mb) and *SPIRE2* (16.04–16.07 Mb). The variant on chromosome 24 was located in the *ZBTB7C* gene (47.8–48.2 Mb) in the goat genome, which could be an ortholog of the *ZBTB17* ‘pigmentation’ gene. However, *ZBTB17* occurs at 50.1 Mb on chromosome 16 of the goat genome. *ZBTB7C* is involved in the negative regulation of cell proliferation (NCBI, 2023).

The three other genes, *THSD7B*, *MAPRE1*, and *GAS8,* that showed significant variants were not directly related to coat color but were described to have roles in cell differentiation and developmental processes, such as cytoskeleton reorganization (*THSD7B*), microtubule polymerization or depolymerization (*MAPRE1*), and cell motility (GAS8) in mammals and fishes. The gene *LOC102181419* has not been extensively characterized. Even though the gene *MC2R* is listed in the pigmentation catalog, there is little evidence to suggest that it is associated with immediate coat color variation.

## 4 Discussion

### 4.1 Phenotypic diversity

In the current study, the phenotype diversity appeared to be significant over regions and locations, with more distinct differences observed at the regional level (Table 2). The Outlier populations (UBR, GGS) showed the highest (for cashmere length, yield, and diameter) and the lowest (for cashmere percentage) mean values.,. It supports the historic breeding practices which involved Russian goat breeds to improve cashmere yield of local goats (Takahashi et al., 2008). The phenotypic diversity of all populations suggested morphological differentiations at the regional level, which were reflected in their slightly differentiated and clustered origins. The Mongolian landscape covers a vast area of diverse natural zones, including the high mountainous zone (west), forest-steppe (Khangai, or north), lower plain steppes (east), and the Gobi Desert (south, southwest), which differ in ecological, climatic, and geophysical conditions (Yembuu, 2021). The populations sampled in the present study were from distinct ecological regions. TSK, GLU and BDU are located in the lower plain steppe in the east. Populations of BUU, TEU, and ZJE are located in the forest steppe in the Khangai region, while Erchim Khar is from the Khuvsgul mountain region in the north. ULU and ZBL are located in the northern part of the West region, where The Great Lakes Basin characterizes the environment. ATU, KHU, and MSK are distributed in the southwest region, where the Mongol Altai and Gobi Altai mountains stretch from west to south. GGS is located in the south of the country, where the Gobi Desert covers most of the area. UBR is located in the western-most part of the country, closest to the Khuiten Peak, the highest peak in the country.

### 4.2 Genetic structure and diversity

The dispersing behavior of populations GGS and UBR was expected as they are believed to have been admixed in the past with Russian goats, where GGS is admixed with Pri-Don and UBR with Gorno-Altay (Samdanjamts and Minjigdorj, 2016). Mukhina et al. (2022) included five Mongolian cashmere goat breeds, of which four populations had the same origin as those presented in our study (ZBL as Buural, ERK as Erchim, GGS as Gobi GS, and ULU as Ulgii Red) and showed different results (Mukhina et al., 2022). The populations from Erchim (the same as ERK in our study) and Dorgon (not included in our study) showed the most differentiated clusters, whereas ULU, ZBL, and GGS clustered together. This difference could be caused by many factors, including different SNP chips (Goat 50K BeadChip by Illumina Inc.), different filtering steps (pruning and QC), or discrepancies in sampling, where different samples from the same population - potentially from different sources - might have been chosen, leading to variation in representation. When clustered with other Asian goat breeds, GGS tended towards the Orenburg breed from Russia, suggesting an exotic admixture (Mukhina et al., 2022). Comparing PCA-based differentiation to international goats, five indigenous Ugandan goat populations also showed small differences, with PC1 and PC2 at 3.6% and 2.9%, respectively (Onzima. et al., 2018), while six Chinese goat breeds showed 11.19% (PC1) and 8.63% (PC2) (Berihulay et al., 2019). The cumulative genomic variance across continents was 22.02% (PC1–13.09%, PC2–8.93%) when obtained from 144 goat breeds worldwide (Colli et al., 2018). Relatively low values of principal components in Mongolian cashmere goats could be explained by low selection pressure and pastoral production system where breeding buck were often exchanged between regions (personal communication).

Overall, the *Fst* results of this study were in line with those of Mukhina et al. (2022), who reported weak differentiation among five Mongolian goat populations, ranging between 0.009 and 0.035. Previous studies have also observed low differentiation among Mongolian goat populations (Nyamsamba et al., 2002; Takahashi et al., 2008; Ganbold et al., 2020).

Italian goat breeds had pairwise *Fst* ranging between 0.013 and 0.164 (Nicoloso et al., 2015) while Pakistani goats show pairwise *Fst* between 0.011 and 0.192 (Muner et al., 2021). A study covering 144 goat populations worldwide reported Wright’s fixation index ranging from 0.00 (two goat populations in Uganda) to 0.556 (Icelandic goat and Manica goat in Mozambique) (Colli et al., 2018). They observed weak differentiation among pastoral management systems, such as in populations from southern Italy and Africa, similar to the Mongolian livestock management system.

For different species, sheep breeds have an average *Fst* of 0.04–0.282 (O’Brien et al., 2020), and Spanish beef cattle breeds have a *Fst* of from 0.026 to 0.068 (Cañas-Álvarez et al., 2015). Hall (2022) compared Wright’s differentiation indexes obtained on microsatellite and SNP markers among different species and suggested benchmark values for each species and marker type, to indicate ‘breed-level’ differentiation within species. For goats, the study suggested *Fst* of 0.08–0.16 and 0.04–0.14 for SNP and microsatellite markers, respectively. Considering this benchmark, none of the studied goat populations were differentiated at the breed level.

This relatively low differentiation index can be explained by the country’s traditional pastoral management system as well as the sudden increase in the goat population since the 1990s. Additionally, herders tend to exchange breeding bucks over long distances to improve cashmere production, according to an unpublished survey by Purevdorj et al. (2018). For example, 10 out of 17 herders in Erdeneburen soum, Khovd Province bought breeding bucks from as far as Dornod Province (east of the country) or from other neighboring provinces. The trading of breeding animals has been encouraged by Regulation No: A/46, which has been in effect since 1998 (GoM, 1998), to support the breeding of highly productive animals. Since 2022, arbitrary animal trading has officially stopped owing to animal disease outbreaks and COVID-19 (GoM, 2022); however, unofficial trade may still occur. This change di`d not affect the results of this study, as all animals in this study were sampled in 2020.

### 4.3 Inbreeding levels based on runs of homozygosity

The assumption that one recombination event happens per generation per chromosome (1.0 cm/Mb) suggests the abundance of different lengths of ROH segments reflects the historical inbreeding levels in a given population (Curik et al., 2014). Thus, it is assumed that longer ROH segments are genome traces that come from the recent same ancestry and shorter ROH segments are genomic traces of earlier ancestry, whose genome is broken into smaller segments over many generations (Curik et al., 2014).

GGS showed the largest number of small ROH segments compared to other populations, which could reflect its selective breeding activity occurred during the 1960s. This breeding practice was an effort to formulate a ‘goat breed’ selected for ‘cashmere production’; thus, GGS was registered as the first goat ‘breed’ in 1971 (Samdanjamts and Minjigdorj, 2016). It has been reported that GGS has the most variable ROH lengths, with longer ROHs than other populations (Mukhina et al., 2022). In a previous study, GGS showed the largest *F*_*ROH*_ (0.023) at 1Mb minimum length among five Mongolian goat populations, and *F*_*ROH1*_ ranged between 0.007 and 0.019 for the other four (Mukhina et al., 2022). In the present study, *F*_*ROH1*_ was 0.029 for GGS, which was a comparable with the previous study.

The 50K SNP panel revealed *F*_*ROH2*_ ranging from 0.008 to 0.024 for indigenous Ugandan goats (Onzima et al., 2018). In Swiss goat breeds mean *F*_*ROH1*_ ranged between 0.033 and 0.09, while in goat breeds in Egypt, *F*_*ROH1*_ ranged from 0.02 (Barki indigenous goat) to 0.09 (Boer) (Burren et al., 2016; Kim et al., 2016). A study considering nine goat breeds from Canada and Australia with different production purposes, using no fixed ROH size threshold, identified a mean *F*_*ROH*_ ranging from 0.009 (rangeland) to 0.057 (Boer and Nubian, Canada) (Brito et al., 2017). In our study, *F*_*ROH2*_ ranged from 0.007 (GLU) to 0.048 (UBR), relatively low compared to other international breeds while comparable to indigenous goats in Uganda and Egypt, maybe due to similar extensive production system. Cashmere goats in Inner Mongolia, China showed *F*_*ROH*_ of 0.026 and 0.071 (Zhao et al., 2024), the difference could be caused by stronger selection pressure.

The overall low inbreeding coefficients obtained in this study could reflect the mobile lifestyles of the herders (Erdenebaatar and Humphrey, 1996), or limited use of artificial insemination and a low selection pressure. Similar to the low differentiation index, an increase in the goat population and frequent exchange of breeding bucks between provinces contributed to the overall small inbreeding.

The limitation of detecting ROH segments with medium density marker is that some apparent ROH segments smaller than 4 Mb may be false positives (Ferenčaković et al., 2013). Additionally, different approaches for estimating ROH segments and non-standardized parameter sets make the number of ROH segments and *F*_*ROH*_ observations not directly comparable; however, they are well suited to detect populations’ past and recent inbreeding levels (Curik et al., 2014; Peripolli et al., 2017). The two different approaches showed the Wahlund effect, which is expressed by a deficiency of heterozygosity in a substructured population (Wahlund, 1928). Thus, the latter approach has more power to express heterozygosity estimates at the subpopulation level. For global goats, mean H_E_ and H_O_ estimates were 0.356 and 0.366, respectively, (Colli et al., 2018), while our study revealed the average H_E_ and H_O_ in Mongolian cashmere goats to be 0.386 and 0.380, respectively. Slightly larger heterozygosity values could indicate underrepresented genomic diversity.

### 4.4 GWAS

Given that coat color is a binary trait with only two phenotype types, case and control, the SNP-based heritability estimates for coat color cases were all close to 0.99 (Yang et al., 2011). The body weight heritability estimate using pedigree data was 0.35 and 0.47, respectively for Angora goats from two different stations (Snyman and Olivier, 1999). For Raeini cashmere goats in Iran, body weight heritability ranged from 0.22 to 0.32, from birth to 12 months of age (Mohammadi et al., 2012). The SNP-based heritability estimates for birth, weaning, and yearling weights were 0.11, 0.27, and 0.10, respectively for Inner Mongolian cashmere goats (Zhang et al., 2021).

Heritability estimates for fleece weight and fiber diameter in Angora goats ranged from 0.22 to 0.30 (Snyman and Olivier, 1999). For cashmere goats originating from several countries (Scotland, Iceland, Tasmania, New Zealand, and Siberia), the heritability obtained for fiber diameter and fiber length, were 0.63 and 0.49, respectively (Bishop and Russel, 1996). Pedigree-based heritability estimates for down hair weight, diameter, and length in Australian cashmere goats were 0.61, 0.47, and 0.70, respectively (Pattie and Restall, 1989). Our results on cashmere trait heritability (ranging from 0.68 to 0.77) could be inflated, but from pedigree analysis, cashmere traits showed relatively high heritability values reported by abovementioned studies.

The ‘inflated’ $\hat{h}$ ^2^_SNP_ estimates of 0.78 for body weight in the current dataset could most likely be caused by the following factors: first, the sample size was too small compared to the suggested number of 3,160 unrelated animals needed for GCTA to obtain a small standard error (SE < 0.1) (Yang et al., 2011). The 537 animals were not pruned by relatedness because of their small sample size. Thus, the relatedness of animals inflates the $\hat{h}$ ^2^_SNP_ because of the relatives’ genetic correlation (Yang et al., 2017). In terms of the independence of the markers, they were also not pruned by linkage disequilibrium, which contributes to the inflation of $\hat{h}$ ^2^_SNP_ (Krishna Kumar et al., 2016). Moreover, the dataset consisted of a range of subpopulations with population stratification. When a population is stratified, the GCTA creates a highly skewed GRM, which eventually leads to an inflated heritability estimation (Krishna Kumar et al., 2016).

An association study on body conformation traits (body weight, body height, body length, heart girth, etc.) in Pakistani goats identified several variants, with the most significant variants on chromosomes 8 and 16 based on a 50K SNP array (Moaeen-ud-Din et al., 2022). Zhang et al. (2021) reported 21 significant genome-wide variants related to body weight traits on chromosomes 6, 7, and 25. In their study, the genes *MAPK3*, *ADGRE2*, and *LDB2* have been suggested to be associated with birth, weaning, and yearling body weight in Inner Mongolian cashmere goats. A GWAS of body weight traits in young (4-month-old) Karachai goats in Russia suggested that the following most putative genes: *MSTN*, *HEG1*, *FGF10*, *FGF14*, *GHRH*, and *SLAIN2* (Selionova et al., 2022). In the same Karachai goats, at 8 months of age, *CRADD*, *HMGA2*, *MSRB3*, *MAX*, *HACL1,* and *RAB15* are suggested to be most significantly associated with body weight (Easa et al., 2022). An SNP in *IGF-1* (insulin growth factor −1) was associated with body weights at different time points (birth weight, 6-month-old, and 12-month-old) in Nanjiang Huang goats (Zhang et al., 2008).

For cashmere traits, *FGF12*, *SEMA3D*, *EVPL*, and *SOX5* have been significantly related to skin and hair growth, and genes such as *GALNTL5*, *FBF1*, *SPHKAP*, and *RGS12* have been suggested to be associated with fleece traits in Inner Mongolian cashmere goats (Wang et al., 2021). *POU1F1*, *MREG*, *ADGFRV1*, and *DUOX1* have been related to mohair quality, mohair volume, grease percentage, and yearling fleece weight, respectively, in Markhoz goats (Nazari-Ghadikolaei et al., 2018). A selection signature study of Inner Mongolian cashmere goats suggested *WNT10A* and *CSN3* as potential genes linked to cashmere trait candidates (Jin et al., 2020). A study using the weighted gene co-expression network analysis identified 10 genes (*WIF1*, *WNT11*, *BAMBI*, *FZD10*, *NKD1*, *LEF1*, *CCND3*, *E2F3*, *CDC6*, and *CDC25A*) with regulatory roles in hair follicle development in Inner Mongolian cashmere goats (Gong et al., 2022).

In our study, we detected signals on chromosomes 2, 4, 5, 9, and 15, where some of the previously identified candidate genes for cashmere traits are located. For example, the *SHKAP* gene is located on chr2:19.4–19.6 Mb, while *SOX5* and *WIF1* are located on chromosome 5 in the goat genome. A significant variant observed on chromosome 4 for cashmere yield was in the proximity of the gene *GALNTL5* (chr4:5.34–5.39 Mb) and has a role in fleece traits in Inner Mongolian cashmere goats (Wang et al., 2021). Another significant variant observed on chromosome 15 for cashmere length was located approximately 2 Mb downstream of the *ALX4* gene (chr15:8.62–8.64Mb), which is a candidate gene for hair follicle development (Qiao et al., 2017).

As quantitative traits are polygenic by nature, the chance of finding truly associated variants increases with a larger sample size (>1000 or >10,000) and a larger number of markers (Visscher et al., 2012). The most reliable results for polygenic traits are usually obtained using over 100,000 samples (Visscher et al., 2012). Thus, one main limitation of our study was its small sample size. In terms of the significance level, using a threshold that is too stringent could have lowered the detection of potentially significant variants. From a technical perspective, recording errors can occur during phenotype measurements, which can affect the detectability of causal variants. To obtain more accurate results from the current dataset, alternative methods, such as imputed haplotypes, can be considered to further investigate the potential causative regions for quantitative traits.

Coat color phenotypes in Markhoz goats have been studied, with the most significant variants located on chromosomes 6 and 13 (Nazari-Ghadikolaei et al., 2018). Significant variants on chromosome 6 have been associated with white color, whereas those on chromosome 13 have been associated with black and brown colors. Further, the identified significant variants are located within or near regions of *ASIP*, *ITCH*, *AHCY*, *RALY*, *KIT*, and *PDGFRA* genes, potentially causing the main coat color diversity in Markhoz goats (Nazari-Ghadikolaei et al., 2018). Another type of variation, two copy number variations (CNVs) downstream of the *KIT* gene on chromosome 6 and four CNVs in the *ASIP* gene on chromosome 13 are associated with white, white-spotted, or tan phenotypes in Pakistani goats (Henkel et al., 2019).

Fontanesi et al. (2009) suggested that CNVs in the *ASIP* gene might cause white coat color in Girgentana and Saanen breeds in Italy. Henkel et al. (2021) confirmed that the presence and absence of CNVs in *ASIP* are responsible for the solid white, greyish, black neck, and copper neck phenotypes in Swiss goats. Another study on French Saanen goats identified significant SNPs on chromosomes 4, 5, and 13 linked to the ‘pink-neck’ and ‘pink’ phenotypes. They observed the strongest signal for coat color at position 61.7 Mb near the *ASIP* gene on chromosome 13 (Martin et al., 2016).

Ganbold et al. (2019) identified seven haplotypes formed by five SNPs on the *MC1R* gene, distributed at approximately 16.05 Mb on chromosome 18, in Mongolian goats. In our study, the two SNPs identified were located on 16.15 and 16.19 Mb on chromosome 18. As we could not locate the *MC1R* gene in the current assembly of the goat genome, we used the *MC1R* gene in the cattle genome as the reference, as, barring a few exceptions, cattle and goat genome have high similarity in karyotype structure (Berardino et al., 2001). The *MC1R* gene is located at 14.7 Mb on chromosome 18, in the cattle (*Bos taurus*) genome and is a known pigmentation gene that causes color phenotype diversity in various cattle breeds (Rouzaud et al., 2000; Park et al., 2012; Schmutz and Dreger, 2013; Matsumoto et al., 2020) and other species (Newton et al., 2000; Andersson, 2003; Chen et al., 2019). Thus, we speculated that the *MC1R* gene or extension locus on chromosome 18 may be the main gene associated with coat color variation in Mongolian cashmere goats.

A review on family genes of *MCR* concluded that while *MC1R* has been studied extensively and plays a significant role in melanogenesis, other genes have roles in glucocorticoid secretion (*MC2R*), energy control (*MC3R* and *MC4R*), and various physiological processes (*MC5R*) (Switonski et al., 2013).

Another resource used as a reference for coat color genes was a ‘pigmentation’ gene catalog by Baxter et al. (2019), on the website (http://www.ifpcs.org/colorgenes/). This catalog contains over 600 genes related to pigmentation and color phenotypes in three species: humans, mice, and zebrafish. To track down the ‘pigmentation genes’ near the observed variants, all candidate genes from the catalog were checked for their locations in the goat genome.

Considering the possible paralog presence of genes and different types of genomic variants causing coat color differentiation, the detection of true causal variants using SNP markers is somewhat limited. However, we found significant variants in the same chromosomal regions (mostly on chromosomes 6, 13, and 18), similar to previous GWAS studies on goat coat color. The two most significant SNPs were observed on chromosome 18, which is close to the location of *MC1R* (Ganbold et al., 2019). These two variants are highly significant for the white phenotype of Mongolian cashmere goats.

Our current observation is based on the physical locations of the significant variants alone. Consequently, it is subject to bias from LD, as genotyped SNPs may be in high LD with true but ‘ungenotyped’ causal variants (Visscher et al., 2012). Moreover, the physical locations of the SNPs (map file) produced by Axiom may not be in line with the current genome assembly map in the NCBI database.

A limitation of the current dataset is the lack of detailed information on animal coat color combinations. A more comprehensive recording of phenotypes could help detect true causal variants that affect the coat color phenotype. Additionally, further investigations at the gene expression level would help understand the functions of potential causal variants and their possible interactions to form myriad colors in goats and other animals.

## 5 Conclusion

Mongolian cashmere goats show significant regional phenotypic diversity in body weight and cashmere quality traits. In terms of the genetic structure, the studied populations showed very small differences. Inbreeding levels were low for all populations except the UBR. This may be due to the traditional transhumance lifestyle and the frequent exchange of breeding animals, even over long distances. The genetic clustering identified two goat populations with the largest exotic admixture (UBR and GGS) and other ‘local’ populations with a small admixture of added ancestry at k = 2. GWAS for quantitative traits identified the strongest signal variant on chromosome 4 for cashmere yield. GWAS of coat color variations indicated that the most significant variants on chromosomes 6, 13, and 18 were likely associated with *KIT*, *ASIP*, and *MC1R* genes.

Overall, this study revealed the phenotypic diversity of production traits and population genetic structure of Mongolian cashmere goats. To increase the power of the GWAS results, a larger number of SNP chips and sample size could be considered. Furthermore, the inclusion of the current dataset into broader collections of goat populations would help identify genomic variants specific to Mongolian cashmere goats.

## Data Availability

The original contributions presented in the study are publicly available. This data can be found here: https://datadryad.org/stash/dataset/doi:10.5061/dryad.sqv9s4ncc.
