# Supplementary material for: Genetic characterization of cashmere goat (Capra hircus) populations in Mongolia
Source: Front Genet. 2024 Sep 17;15:1421529. doi: 10.3389/fgene.2024.1421529 (PMC11442248; doi:10.3389/fgene.2024.1421529)
Supplement: Supplementary file 1 [file Table1.DOCX]

# Supplemental Material

Table 1. Population sizes, expected (H_E_) and observed (H_O_) heterozygosity for each population.

|  | Population | N | H*_E_* ± SD | H*_O_* ± SD |
| --- | --- | --- | --- | --- |
| 1 | ATU | 90 | 0.386 ± 0.0003 | 0.376 ± 0.011 |
| 2 | BDU | 71 | 0.386 ± 0.0003 | 0.374 ± 0.014 |
| 3 | BUU | 91 | 0.386 ± 0.0003 | 0.382 ± 0.013 |
| 4 | ERK | 87 | 0.386 ± 0.0004 | 0.384 ± 0.013 |
| 5 | GGS | 92 | 0.386 ± 0.0002 | 0.390 ± 0.013 |
| 6 | GLU | 89 | 0.386 ± 0.0001 | 0.379 ± 0.005 |
| 7 | KHU | 89 | 0.386 ± 0.0002 | 0.379 ± 0.008 |
| 8 | MSK | 70 | 0.386 ± 0.0004 | 0.381 ± 0.017 |
| 9 | TEU | 64 | 0.386 ± 0.0003 | 0.386 ± 0.010 |
| 10 | TSK | 81 | 0.386 ± 0.0004 | 0.372 ± 0.020 |
| 11 | UBR | 26 | 0.386 ± 0.0002 | 0.379 ± 0.028 |
| 12 | ULU | 86 | 0.386 ± 0.0002 | 0.376 ± 0.009 |
| 13 | ZBL | 93 | 0.386 ± 0.0003 | 0.378 ± 0.013 |
| 14 | ZJE | 77 | 0.386 ± 0.0003 | 0.384 ± 0.012 |
| Overall mean | | | 0.386 ± 0.0003 | - 1. ± 0.014 |


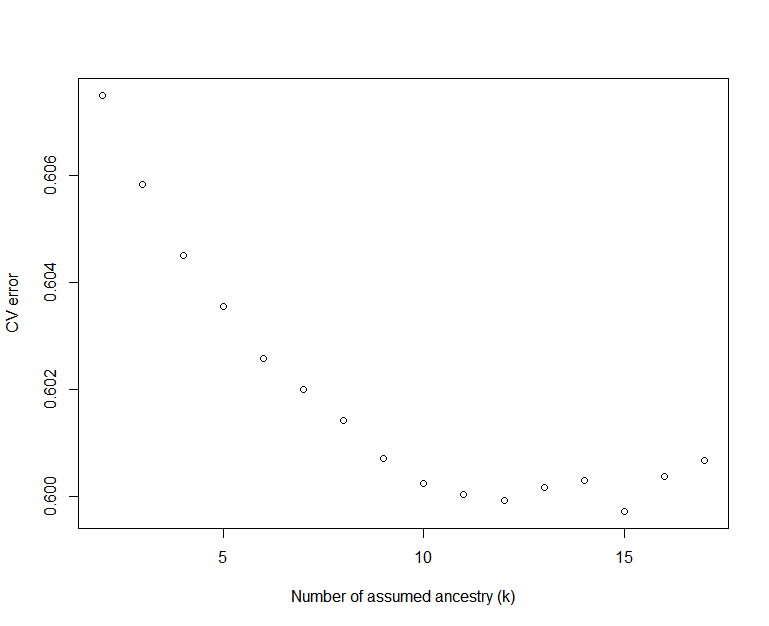


Figure 1. Cross-validation error plot.


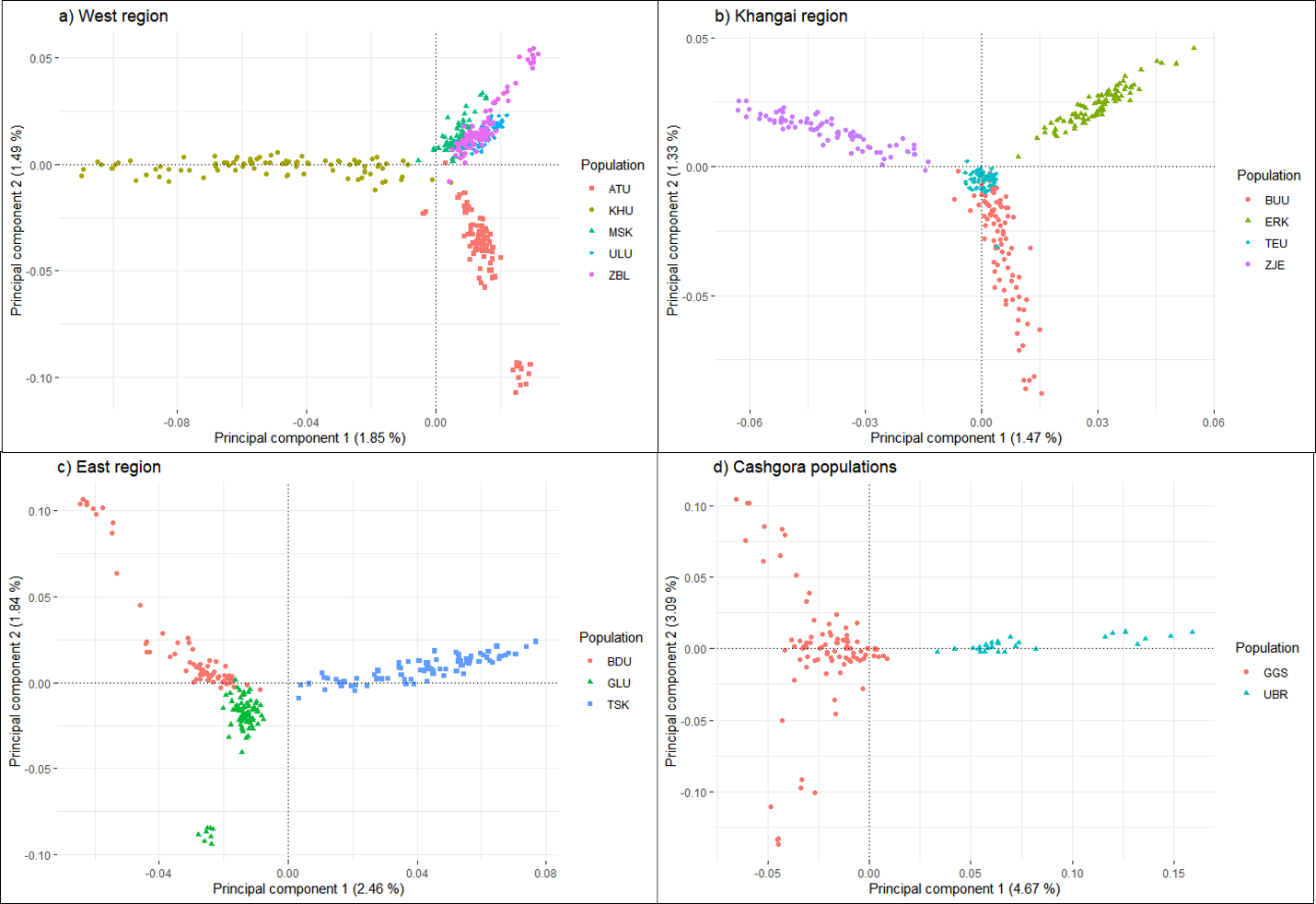


Figure 2. PCA plot within regions

Table 2. Expected and observed heterozygosity, SNP based inbreeding value (F)

| **Population name** | **N** | **Taken as one population** | | | **Taken separately** | | |
| --- | --- | --- | --- | --- | --- | --- | --- |
|  |  | ***H_E_*** | ***H_O_*** | ***F*** | ***H_E_*** | ***H_O_*** | ***F*** |
| ATU | 90 | 0.3857 ± 0.0003 | 0.376 ± 0.011 | 0.024 ± 0.030 | 0.3965 ± 0.0121 | 0.3933 ± 0.00026 | -0.0083 ± 0.031 |
| BDU | 71 | 0.3857 ± 0.0003 | 0.374 ± 0.014 | 0.029 ± 0.035 | 0.3970 ± 0.0143 | 0.3943 ± 0.00027 | -0.0068 ± 0.036 |
| BUU | 91 | 0.3858 ± 0.0003 | 0.382 ± 0.013 | 0.009 ± 0.034 | 0.3994 ± 0.0140 | 0.3986 ± 0.00020 | -0.0020 ± 0.034 |
| ERK | 87 | 0.3857 ± 0.0004 | 0.384 ± 0.013 | 0.005 ± 0.034 | 0.4008 ± 0.0137 | 0.3970 ± 0.00031 | -0.0096 ± 0.035 |
| GGS | 92 | 0.3857 ± 0.0002 | 0.390 ± 0.013 | -0.012 ± 0.034 | 0.4035 ± 0.0136 | 0.4027 ± 0.00021 | -0.0019 ± 0.034 |
| GLU | 89 | 0.3858 ± 0.0001 | 0.379 ± 0.005 | 0.018 ± 0.013 | 0.3973 ± 0.0050 | 0.3952 ± 0.00012 | -0.0052 ± 0.013 |
| KHU | 89 | 0.3858 ± 0.0002 | 0.379 ± 0.008 | 0.017 ± 0.020 | 0.3986 ± 0.0078 | 0.3945 ± 0.00017 | -0.0102 ± 0.020 |
| MSK | 70 | 0.3857 ± 0.0004 | 0.381 ± 0.017 | 0.012 ± 0.046 | 0.3997 ± 0.0183 | 0.3948 ± 0.00032 | -0.0124 ± 0.047 |
| TEU | 64 | 0.3857 ± 0.0003 | 0.386 ± 0.010 | -0.0016 ± 0.027 | 0.4019 ± 0.0108 | 0.3989 ± 0.00024 | -0.0076 ± 0.028 |
| TSK | 81 | 0.3857 ± 0.0004 | 0.372 ± 0.020 | 0.035 ± 0.053 | 0.3952 ± 0.0216 | 0.3938 ± 0.00031 | -0.0036 ± 0.055 |
| UBR | 26 | 0.3857 ± 0.0002 | 0.379 ± 0.028 | 0.016 ± 0.073 | 0.4011 ± 0.0296 | 0.3933 ± 0.00024 | -0.0196 ± 0.075 |
| ULU | 86 | 0.3858 ± 0.0002 | 0.376 ± 0.009 | 0.026 ± 0.023 | 0.3965 ± 0.009 | 0.3956 ± 0.00017 | -0.0023 ± 0.023 |
| ZBL | 93 | 0.3857 ± 0.0003 | 0.378 ± 0.013 | 0.020 ± 0.034 | 0.3991 ± 0.0134 | 0.3961 ± 0.0002 | -0.0076 ± 0.034 |
| ZJE | 77 | 0.3857 ± 0.0003 | 0.384 ± 0.012 | 0.006 ± 0.033 | 0.4002 ± 0.013 | 0.3972 ± 0.0002 | -0.0075 ± 0.033 |
